# Vec4Cred: a model for health misinformation detection in web pages

**DOI:** 10.1007/s11042-022-13368-z

**Published:** 2022-07-28

**Authors:** Rishabh Upadhyay, Gabriella Pasi, Marco Viviani

**Affiliations:** Department of Informatics, Systems, and Communication, University of Milano-Bicocca, Edificio U14 – ABACUS, Viale Sarca, 336, Milan, 20126 Italy

**Keywords:** Health misinformation, Consumer health, Natural language processing, Machine learning, Deep learning

## Abstract

Research aimed at finding solutions to the problem of the diffusion of distinct forms of non-genuine information online across multiple domains has attracted growing interest in recent years, from opinion spam to fake news detection. Currently, partly due to the COVID-19 virus outbreak and the subsequent proliferation of unfounded claims and highly biased content, attention has focused on developing solutions that can automatically assess the genuineness of health information. Most of these approaches, applied both to Web pages and social media content, rely primarily on the use of handcrafted features in conjunction with Machine Learning. In this article, instead, we propose a health misinformation detection model that exploits as features the embedded representations of some structural and content characteristics of Web pages, which are obtained using an embedding model pre-trained on medical data. Such features are employed within a deep learning classification model, which categorizes genuine health information versus health misinformation. The purpose of this article is therefore to evaluate the effectiveness of the proposed model, namely Vec4Cred, with respect to the problem considered. This model represents an evolution of a previous one, with respect to which new features and architectural choices have been considered and illustrated in this work.

## Introduction

The problem of widespread online misinformation has given impetus, in recent years, to the proliferation of several research proposals aimed to curb this phenomenon from different perspectives and with respect to distinct application domains. The proposed approaches have been applied mainly to fake review detection in review sites, i.e., online platforms that allow users to publish reviews about products and services [41], and fake news detection in microblogging platforms, which often disseminate newsworthy content related to useful public information, such as politics and general-interest events [64]. The majority of these solutions are based on the identification of particular characteristics (i.e., *features*) that are highly domain-specific, related to the content being disseminated, the purpose of the dissemination, the considered platform, the authors of the content, and possible social interactions in the case of social networking sites. These features are, in such approaches, employed within supervised classifiers, which categorize information into genuine and non-genuine, using “standard” Machine Learning or more recent deep learning solutions [20, 59].

In this context, a domain that has most recently attracted the attention of researchers is that of health information disseminated online. In fact, when searching for possible health treatments or advice, one might incur in a severe harm if finding fake or inaccurate content, the so-called *health misinformation* [9]. In most cases, people who are not an expert in the field are unable to properly assess the reliability of health-related claims, both, in general, due to their *limited cognitive abilities* [39] and, more specifically, due to their insufficient level of *health literacy* [55]. The difficulties in providing laypeople with automated solutions to compensate for the complexity of evaluating health information on their own, in an online context that is less and less mediated by the presence of medical experts [13], lie in the fact that content related to health is now generated online in large quantities and at very high speed (which makes it difficult even for experts to assess misinformation) and since it has its own peculiarities compared to other domains of interest. First of all, both long and semi-structured texts published on “traditional” Web pages (e.g., forums, blogs, question-answering medical systems, etc.), and very short and unstructured texts spread through microblogging platforms (e.g., the mass of COVID-19-related tweets in the last year) are diffused online. Secondly, these texts can be characterized both by a scientific language and possible reference to external resources that can be taken into account when assessing their genuineness, or by the use of more informal language that generally characterizes user-generated content.

Given the magnitude of the problem, in this work we focus on health-related content disseminated in the form of Web pages, an area in which research has mainly identified some *handcrafted features* that make a site or a page “reliable”, through the use of user-based studies or ML approaches. These approaches have been shown not to be particularly effective, or just for specific scenarios, since the choice of features to consider and design is highly domain-dependent [58]. To overcome such issues, with respect to the literature, we explore the possibility of representing Web pages by means of automatically learned embedding features by taking inspiration from Web2Vec, a solution recently proposed for phishing Web page detection [15]. Unlike that model, in the proposed solution, i.e., Vec4Cred, we inject some health-related genuineness factors in the feature extraction phase and in the deep learning architecture used, which also consider linguistic aspects not previously taken into account. In particular, Vec4Cred extends our previous work presented in [58] but, compared to that work, the model proposed in this paper investigates new semantic aspects in the automatic feature extraction phase and new architectural configurations. Evaluations are performed against publicly available datasets containing health-related content in the form of Web pages, labelled w.r.t. their genuineness.

The rest of the article is organized as follows. Section 2 provides background concepts and an overview on the existing theoretical and automated approaches for misinformation detection in Web pages in the health domain. Section 3 describes the proposed Vec4Cred model and the reasons for the modeling and implementation choices made. Section 4 illustrates the experimental evaluations performed to assess its effectiveness. Finally, Section 5 summarizes the contribution of the work and discusses further research directions.

## Background and related works

In this section, we focus primarily on describing background concepts related to health misinformation, health literacy, and specific approaches that have been proposed in the last decade for health misinformation detection in Web pages, mentioning, however, some recent research directions with respect to social media platforms.

### Health misinformation and health literacy

According to [56], *health misinformation* can be defined as “a health-related claim that is based on anecdotal evidence, false, or misleading owing to the lack of existing scientific knowledge”.^1^ As part of the assessment of such claims, it is necessary to take into account some specific characteristics of the health domain. Health information (both on dedicated Websites and in social media), is characterized by great *variability* with respect to distinct aspects, such as the *topics* discussed, the employed *vocabulary*, and the *linguistic register* used. Topics can be referred to diseases, therapies, vaccines, medications, health treatments, wellness, etc. This has a direct impact on the employed vocabulary, which may change with respect to use of specific terms relating to different medical problems. Furthermore, health information is characterized by a given *degree of depth* and a *target audience* [45]. This means, on the one hand, that we can find information designed for the general public, which must be as comprehensible as possible (this can lead to over-simplification); on the other hand, that we can find professional content that is aimed at an audience of researchers and scholars (practically incomprehensible by the average user).

Faced with such heterogeneity, people can find it difficult to manage health information correctly, first, due to their *limited cognitive abilities* [39], as briefly introduced in Section 1, and, secondly, due to a lack of a sufficient level of *health literacy*. This latter concept was included in the glossary of the *World Health Organization* (WHO) in 1988 [28], and indicates the ability of a citizen to obtain, process, and understand basic health information in order to make informed choices. The study of health literacy rates is a particularly important topic especially in welfare and, over the years, has attracted steadily increasing interest especially at the level of institutions and government agencies [55]. Increasing health literacy rates in the population, including through the development of automated tools, therefore becomes crucial in the current scenario of health misinformation diffusion, since in the vast majority of cases laypersons are called upon to play an active role in managing their own health and that of others, as seen recently in the case of the COVID-19 pandemic [55].

In this scenario, several solutions have been proposed in recent years to assess the genuineness of health-information circulating on Websites and social media platforms. Before introducing these solutions, it is worth noting that various concepts have been used that are totally or partially overlapping with that of genuineness (which, in generally, we employ as a general term in this article). In the state-of-the-art works detailed in the next section, it is possible to find a reference, among others, to the concepts of *reliability*, *truthfulness*, *trustworthiness*, *credibility*, *veracity*, etc., which can have specific meanings based on whether they refer to the source of information, the information itself, the communication medium through which information is propagated, or other theoretical aspects.

### Manual or pseudo-automated (interactive-based) approaches

Early approaches that focused on the study of health misinformation were mainly manual or pseudo-automated. Such *interactive-based* approaches concerned, in particular, with evaluating Website and Web page *reliability* and/or *credibility*,^2^ by employing interview questionnaires or other interactions with the users. Such approaches, therefore, are based on the users’ perceptions, which are *subjective* and driven by their information needs and other personal/demographic factors [12, 54].

In [29, 48], the authors stated that users’ evaluations of Web content are influenced, in particular, by *source-related*, *content-related*, and *design-related factors*. Such categories of factors can have either positive or negative effects on the users’ perceptions with respect to the credibility of information. For example, source-related factors such as the authority of owners/sponsors have been shown to have a positive effect on the perception of credibility [33], even if, in some cases, users tend not to trust communication that is too “institutional” [61], perceived in particular by younger users as old and “not cool” [42]. Whereas for content-related factors, the consensus among sources has been considered, for example, as a good credibility indicator, although this aspect is strongly influenced by an individual’s perceived knowledge of the topic, as illustrated in [38]. With respect to personal experience and facts, users have shown mixed feelings. Some users evaluate the presence of objective facts in a positive way [61], while others are dissatisfied by them because considered as unbalanced [49]. Design-related factors can be evaluated either positively or negatively: in general, an aesthetically well-maintained and easy-to-navigate site is perceived as credible. On the contrary, it is perceived negatively [35].

The key features and outcomes of the main pseudo-automated approaches illustrated in this section are summarized in Table 1.

**Table 1:** Summary of pseudo-automated approaches

Paper	Health	Source	Participants	Age	Disease	Outcome
	context				Experience	
[33]	Mental health	Web pages	5	N/R	Mental health patients	Comprehensiveness, authoritativeness, trustworthiness, and currency of health information on mental health Web- sites positively affect users’ perceptions w.r.t. message quality
[42]	HIV prevention	Web pages	40	18-24	African female college students	Interactive features, practical advice, and content authored by familiar and trustworthy sources positively affect users’ perceptions w.r.t. message utility
[12]	General health	Web pages	44	Mean = 37	Italian-speaking adults with diffe- rent health literacy	– Adults with low health literacy mentioned less established credibility criteria compared to those mentioned by the group with the higher health literacy
						– However, common credibility criteria for both groups are: medical authorship, identifiable authorship, institutional authorship, and presence of ad- ditional author’s information
[54]	Diet	Web pages	252	N/R	N/R	Health literacy plays an important role in decreasing the effect of health misinformation on the consumer

Despite the useful outcomes of pseudo-automated approaches, the genuineness of online health information is a complex concept involving more than two dozen dimensions that can be subjectively assessed by users. Therefore, in recent years, a number of solutions have been proposed to automatically address this issue in both Web and social media content.

### Automated approaches

One of the earliest significant works in this area is that described in [63], where the authors presented a framework for predicting the so-called *resource quality* (RQ) of medical Web pages, by using an SVM model trained on 750 resources published on Breast Cancer Knowledge Online (BCKOnline) [31]. The authors described RQ as a composition of *reliability* and *relevance*, where reliability is assessed based on quality dimensions such as *accuracy*, *credibility*, and *currency* [62], and relevance is related to the utility of the page for a user searching for a given medical information. The considered features are *categorical features*, i.e., related to the audience (e.g., age, disease stage, etc.), the type (e.g., medical, supportive, and personal), and the subject of the Web page (e.g., treatment-related, therapies-related, etc.), and simple *textual features*, such as title, description, creator, publisher, and access right.

In [53], an automated approach based on SVM for *reliability* prediction of medical Web pages has been proposed. In the approach, the reliability of a Web page is assessed by performing binary classification. The author explored the usage of *link-based*, *commercial*, *PageRank*, *presentation*, and *textual features*. Link-based features are related to some counting of internal and external links (and related properties) in the Web page. Commercial features refer to the presence of commercial terms in the page. PageRank features are related to the relative importance of a Web page computed via PageRank.^3^ Presentation features refer to the clearly in the presentation of content on the page. Finally, textual features are simply defined as normalised word frequency vectors. Two other recent works based on the use of handcrafted features and Machine Learning approaches are those described in [17, 34]. In [34], a Logistic Regression model for assessing the *reliability* of Web pages has been trained on labeled data collected with respect to 13 vaccine-related search queries. *Textual features* are employed in the form of count-based and TF-IDF word vectors. In [17], a replicability study has been conducted on [53], considering two additional datasets made available in [50, 57], and ignoring PageRank features, deemed as not suitable for assessing Website reliability [44].

Solutions that attempt to refer to criteria of reliability of medical information provided by external bodies are those proposed by [1, 5, 11, 30]. In [5], the capability of an automated system to perform the task of identifying 8 HONcode principles on health Websites has been studied.^4^ Distinct Naive Bayes classifiers are trained over a collection of Web pages labeled with respect to the considered criteria. In this approach, Website content is converted into weighted bag-of-words representations. In [1], to confirm the *evidence-based medicine* (EBM) property of a Web page, the treatment described in the Web page is checked against its approval by the US Food and Drug Administration, the UK National Health System, or the National Institute of Care Excellence. Two different feature types are considered for classification: *text-based* and *domain-specific* features, related to JAMA criteria [52].^5^ A number of distinct ML classifiers have been used for the experiments. In [30], the authors have proposed to automate the use of DISCERN.^6^ Five *Hierarchical Encoder Attention-based* (HEA) models (related to 5 DISCERN criteria) are trained on articles related to breast cancer, arthritis, and depression. A *Bidirectional Recurrent Neural Network* (BRNN) layer converts words, sentences, and documents to dense vector representations. Such representations are used for classification using a softmax layer. A knowledge-guided graph attention network named DETERRENT for detecting *health misinformation* has been recently proposed in [11], trained on articles related to diabetes and cancer. It incorporates a Medical Knowledge Graph and an Article-Entity Bipartite Graph, and propagates node embeddings representing Web pages through Knowledge Paths for misinformation classification.

More recently, the interest of the scientific community to detect health misinformation is turning to the use of *deep learning* (DL) solutions, especially with respect to social media content, achieving promising results [3, 47]. From a review of the literature concerning Web pages, we have identified a DL-based solution related to the tangent problem of phishing Web page detection, i.e., Web2Vec [15]. This model focuses on embedded representations of the target Web page URL, content, and DOM structure. The obtained embedding vectors are used by a hybrid CNN-BiLSTM network to extract local and global features, and combined by an attention mechanism strengthening important features. Multi-channel output vectors are concatenated and provided to a classifier for determining the category of the target Web page (i.e., phishing vs non-phishing). In [58], inspired by Web2Vec, we employed the same DL model to detect health misinformation in Web pages. The model has been applied to their source, content, and design factors, together with contextual aspects related to the presence of reliable links, medical-related terms, and other genuineness indicators. The Vec4Cred model presented in this article, as illustrated in the Introduction, is an extension of this latter work.

The key features and outcomes of the main automated approaches illustrated in this section are summarized in Table 2.

**Table 2:** Summary of automated approaches

Paper	Focus	Health context	Dataset	Features	Model(s)
[63]	Quality (Credibility)	Breast cancer	780 Web pages from BCKOnline	34 feature vectors obtained by 8 BCKOnline metadata elements, including title, description, creator, publisher, type, rights, subject, and audience	SMO, Naive Bayes, J48, IB1, ZeroR
[53] and [17]	Reliability	General health	360 Web pages	Link-based features, commercial features, PageRank features, presentation features, word features	SVM
[1]	Quality	Shingles, flu and migraine	246 Web pages	Linguistic and formal features	Multinomial naive Bayes, k-nearest neighbour, SVM, stochastic gradient descent, logistic regression, multilayer perceptron
[47]	Veracity	General health	Health Stack Exchange (3,958 questions and 2,260 tags) question answering dataset	Word embedding features and sentiment features (i.e., polarity and subjectivity)	Neural networks and convolutional neural networks
[30]	Quality	Breast cancer, arthritis, and depression	269 Web pages	BERT and BioBERT features	Random forests and HEA neural networks
[34]	Reliability	Vaccination	259 texts were classified as reliable, and 183 as unreliable	Bag-of-words features	Naive Bayes and logistic regression
[11]	Misinformation	Cancer and diabetes	8368 articles (fake:2084)	Word embedding features	Bidirectional GRU

## Vec4Cred

**Fig. 1:**
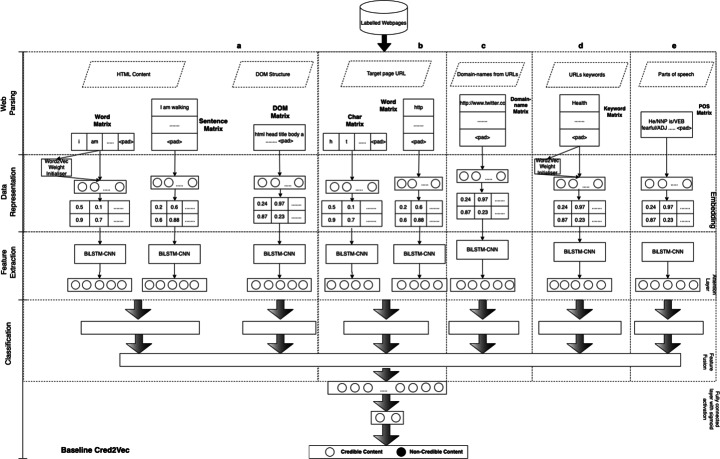
The multi-layer architecture of Vec4Cred. In particular, several configurations of the model are illustrated. In (*a*), only the Web page content and its DOM structure are considered; such information are employed in all the model configurations; (*a*) + (*b*) represents the model configuration in which the URL of the Web Page is also considered, as in the Web2Vec model [15]; (*a*) + (*c*) represents the model configuration proposed in [58], considering the links present in the content of the target Web page; (*a*) + (*b*) + (*c*) is the model configuration in which we add the URLs in the form of domain-names present in the target Web page; with the addition of (*d*), we indicate the model configuration considering also the keyword extracted from the pages referred by the links presents in the target Web Page; finally, the last configuration of the model, represented by the addition of (*e*), considers parts of speech from the target Web Page content

The solution we propose, namely Vec4Cred, is a model for evaluating health misinformation by automatically extracting features from embedding representations of health-related Web page (structural- and content-based) characteristics, and using them in the context of a multi-layer architecture, which is illustrated in Fig. 1. In the Vec4Cred model, some peculiarities related to the problem of assessing the genuineness of health information are taken into account. Foremost, it is considered that the use of a specific vocabulary related to the medical field, when generating an embedded representation of Web pages, is crucial for misinformation detection. In addition, instead of focusing on the characteristics of the single URL of the target Web page (as done in Web2Vec [16]), the URLs present in the content of the page itself are taken into consideration, which can give a better indication of whether they refer to reliable or unreliable external sources (as already done in [58]). To obtain further information by exploiting the URLs present in the target Web page, we also considered the content of the pages referred by such links, and extracted keywords (in an automated way) that were deemed indicators of health information genuineness. Parts of speech were also extracted from the content of the target Web page to incorporate additional information related to grammar aspects.

With the above-mentioned considerations in mind, the proposed model is based on the following four steps (see Fig. 1):

- *Data Parsing*: the Web page content, its Document Object Mode (DOM) structure, its URL, the URLs present in the page, and the content of the pages linked from the target Web page, are parsed from each Web page in the dataset, to extract suitable *word-level* and *sentence-level corpora*, including keywords and parts of speech, that are employed in the following phases;
- *Data Representation*: *word-level embedding representations* are generated for the Web page content, for the keywords automatically extracted from the pages linked in the target Web page, and for the parts of speech extracted from the target Web page; in addition, also *sentence-level embedding representations* are generated for the Web page content. A *word-level embedding representation* is also provided for the DOM structure of the Web page, its URL, and the URLs present in the page;
- *Feature Extraction*: a CNN-BiLSTM network is used to extract features from the given embedding representations;
- *Classification*: a *binary classification* between genuine health information and health misinformation is performed by employing densely connected layers.

Each of the above phases is discussed in detail in the following sections. In particular, several configurations of the proposed model are detailed below, but each consists of the previous four phases.

### Data parsing

The data parsing operation is performed by acting, as introduced before, on the target Web page DOM structure, its content, its URL, and the URLs that are present in the page.

#### DOM structure parsing

HTML files are characterized by a typical semi-structured data format. The hierarchical structure is represented using HTML tags, organized according to the Document Object Model (DOM) structure. Focusing on such a structure, we extracted an ordered list of tags, starting from high-level tags until “children” tags, i.e., HTML, HEAD, META, LINK, TITLE, SCRIPT, BODY, DIV, TABLE, TR, TD, IMG. Such HTML tags are considered as words, which constitute the *world-level corpus* for the DOM structure to be used in the subsequent data representation phase.

#### Web page content parsing

Each Web page is phrased, and only unstructured textual content is considered (links and tags are excluded). Both a *word-level* and a *sentence-level* corpus are constructed. The first is constituted by each distinct word present in the page, the second identifies word sequences separated by the ‘ . ’ character. Specifically, we consider a fixed-length dimension (i.e., around 500 characters, as the average size of word sequences in the dataset) for each word sequence after experimenting with different combination of dimensions.

**Fig. 2:**
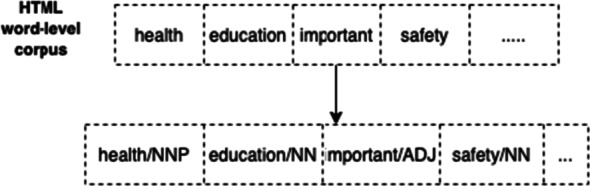
Example of the construction of the POS-level corpus

In addition, *parts of speech* are extracted from the Web page. The choice of considering POS tags associated with words relies on the fact that some work employing text analysis for fake news detection and similar tasks, focused on mining such linguistic information [10, 25, 32, 43]. For example, the work presented in [22] found that fake news often contains huge number of personal pronouns, and other typical grammar characteristics. The parts of speech obtained in our model constitute the POS*-level* corpus that is employed in the following phases of the approach. A simple example of POS tagging is illustrated in Fig. 2.

#### URL parsing

**Fig. 3:**
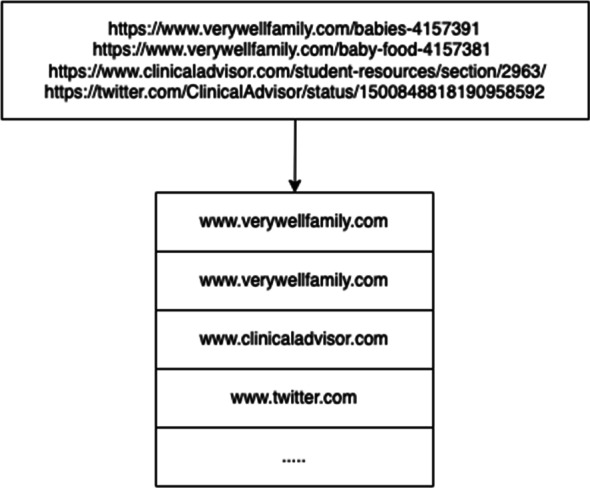
Example of the construction of the word-level corpus from URLs in the target page

The URL of the target Web page and the URLs present in the page are gathered. Then, domain-names are extracted from such URLs in the page. The usage of domain-names as features has been identified as a useful factor for misinformation detection [8, 24, 46]. In fact, when considering the target page, their appearance and succession on the page can be used by the model to learn their association with genuine or non-genuine information. Hence, the sequence of domain-names, an example of which is illustrated in Fig. 3, represent the *word-level corpus* to be employed in the data representation phase.

**Fig. 4:**
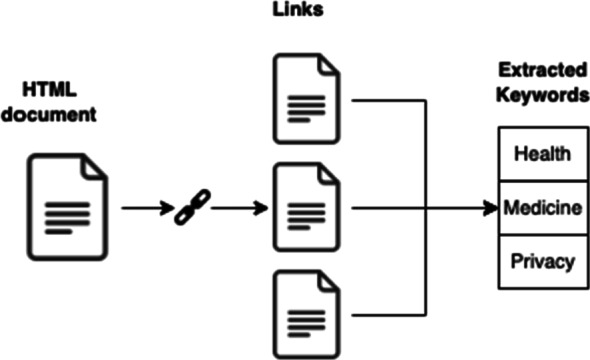
Example of the construction of the word-level corpus for keywords extracted from the linked page content in the target Web page

Furthermore, from the content of the pages associated with the links present in the target Web page, we performed an automatic extraction of keywords. This is motivated by the fact that, in the literature, the fact of referring to some external source has been indicated as potentially useful in terms of information genuineness assessment, at least in some studies [38]. Hence, in this work, we decided to focus on the content of external referenced pages. For keyword extraction, we tested two approaches, one based on the usage of *TextRank* [36], a graph-based text summarization approach, and the other based on the usage of YAKE [6], a statistic-based approach for keyword extraction. We found out that the statistics-based approach was faster and produced better results on a sample of the considered data, where an average of 100 links were referenced in the target Web pages. Therefore, we extracted the top-20 keywords using YAKE for each page referenced by an URL in the target Web page. Such keywords represent the *word-level* corpus to be later employed in the following phases. A simple example of such keyword extraction phase is illustrated in Fig. 4.

### Data representation

In this phase, the word-level corpora related to the Web page DOM structure, content, URLs (URL of the Web page and present in the Web page), keywords extracted from paged linked in the target Web page, and parts of speech extracted from the content of the target Web page, along with the sentence-level corpus related to the Web page content, are formally represented in order to capture their semantic relationships through word embedding. In particular, to perform such data representation, a Keras embedding layer has been employed,^7^ which is based on a supervised method that enhance the representations while training the model using backpropogation [27]. It is worth to be underlined that a separate embedding layer is defined for each data corpus obtained by parsing Web pages, such as the word-level DOM corpus, word-level and sentence-level corpora from Web page content, word-level URL corpora, word-level keyword corpus and POS-level corpus.

**Fig. 5:**
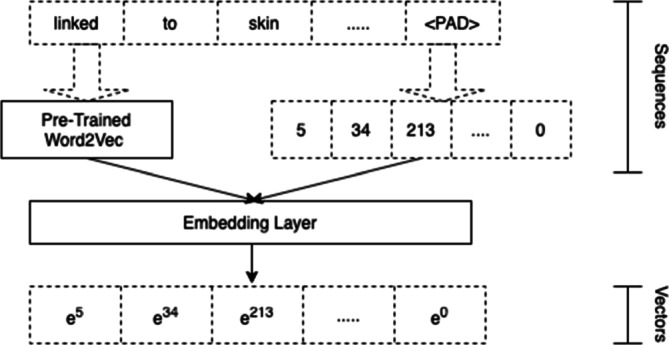
Example of the (Web page content) word-level embedding phase

In Vec4Cred, a *word2vec* [37] layer pre-trained on PubMed is employed as a weight initializer in the Keras embedding layer when considering the Web page content and the word-level keyword corpus, to include domain-specific information related to the medical field.^8^ In this way, the obtained word2vec weights are used as weight initializers for the embedding layer, as done in [58]. An example of such phase is illustrated in Fig. 5.

### Feature extraction

Features are extracted by means of a CNN-BiLSTM network with an attention mechanism applied to the embedding representations obtained in the previous phase. *Convolutional Neural Networks* (CNN)s are nowadays commonly used for local feature extraction from data. In particular, *Bidirectional Long Short-Term Memory* (Bi-LSTM) networks are used to overcome the ability to learn feature from sequences of CNN by combining words with their context [14]. The attention mechanism has been applied to improve the prediction capacity of the model, as illustrated in detail in the following.

#### CNN

The employed CNN is constituted, as in [58], by a feedforward network model structure. The hidden layer is divided into a convolution layer and a pooling layer. To overcome overfitting, each fully connected layer is followed by one dropout layer (with a dropout ratio of 0.05). Details on the convolution and pooling operations can be found in [15]. Using such an architecture, only the important features are extracted.

#### BiLSTM

The output of the CNN layer constitutes the input of the BiLSTM layer. Such layer is formed using *Long Short-Term Memory* in both directions, i.e., forward and backward, which keeps the sequential order among the data. It also allows detecting the relationship between the previous inputs and the output.

Since BiLSTM is a sequential- and memory-based model, it can both learn long-term dependence on the Web page and also extract improved features using local features from CNN. To deal with possible overfitting, *dropout learning* and *L2 regularization* (as detailed in the next section) are used to improve the model training.

#### Attention layer

The addition of the attention layer, in the case of assessing the genuineness of health information, is dictated by the fact that in the same document there may be parts characterized by “more genuine” and “less genuine” information. In this situation, even the presence of a small amount of “non-genuine” features characterizing a genuine page (or vice versa), can negatively affect its final evaluation. The purpose of the attention layer is, therefore, to pay particular attention with respect to the most discriminant features with respect to the considered problem; in this work, we have referred in particular to the concept of *additive attention* [2].

### Web page classification

The last stage of the approach is the categorization of Web pages with respect to their genuineness. In particular, a *binary classification* of pages (i.e., genuine and not genuine) is performed through the use of a classifier consisting of a fully connected layer having a *sigmoid function* in the final layer, which combines the features extracted from the previous layers relating to the six corpora considered (i.e., the word-level DOM corpus, the word-level and sentence-level corpora extracted from the Web page content, the word-level URL corpora, the word-level keyword corpus and the POS-level corpus).

For the classification loss calculation, the *cross-entropy loss function* and the *L2 regularization* are applied to overcome overfitting. Formally:

1 $$ Error(t - y) = - \frac{1}{N}{\sum\limits_{n}^{N}}\left[t_{n}\ln y_{n}+(1-t_{n})\ln (1-y_{n})\right] $$

and

2 $$ Loss=Error(t - y)+\lambda {\sum\limits_{n}^{N}}{w_{n}^{2}} $$

where *t* is the target label, *y* the predicted label, *w* the weight matrix of the layer, and *λ* is the so-called *L2 penalty parameter*.

## Experimental setup

In this section, we present the experimental evaluation of the effectiveness of the proposed model. Specifically, we introduce the description of the different datasets, baselines, and evaluation metrics considered, together with technical and experimental details followed by a discussion on the obtained results.

### Description of the datasets

Only a few publicly available datasets are nowadays currently available for assessing health misinformation. In particular, it has been necessary to consider those datasets from which it was possible to extract the original HTML format of Web pages. Hence, the choice has fallen on the datasets provided by [21, 50, 53], and illustrated in detail in the following. For a comparative evaluation purpose, we also tried to implement the original Web2Vec model as a baseline. However, we could only implement such model for two datasets [21, 50] as the URL associated with each Web page was available only for those datasets.

#### Microsoft credibility dataset

The first considered dataset, provided by [50], is constituted by 1,000 Web pages related to different domains such as Health, Finance, Politics, etc. Web pages are associated with *credibility ratings* provided on a five-point Likert scale, ranging from 1 to 5, where 1 stands for “very non-credible”, and 5 for “very credible”. In [17], for evaluation purposes, labels have been pre-processed by removing the middle values (3 in this case) and mapping 4-5 rating values to credible Web pages and 1-2 rating values to non-credible Web pages. In our approach, we followed the same strategy, and we focused on the 130 health-related Web pages in the dataset, after removing empty or inaccessible Web pages. Out of 130, 104 were credible and 26 were non-credible. Given the high data imbalance, we employed the *Synthetic Minority Over-sampling Technique* (SMOTE) [7] oversampling method to face this problem by oversampling the minority class.

#### Medical web reliability corpus

This is a manually generated balanced dataset with binary labels, i.e., *reliable* and *unreliable*, associated with Web pages [53]. Reliable pages come from randomly selected HON accredited Websites.^9^ Unreliable pages come from Websites searched on the Web by using “unreliable queries”, constituted by the disease name + “miracle cure”. The dataset consists of 360 Web pages, of which 180 reliable and 180 unreliable. After a cleaning phase, to remove blank and no-longer accessible pages, we dealt with 170 reliable and 176 unreliable Web pages.

#### CLEF eHealth 2020 task-2 dataset

This dataset, described in [21], has been expressly created to assess the topical relevance, readability, and *credibility* of Web pages consisting of medical content, as part of the so-called *Consumer Health Search* (CHS) task.^10^ Credibility ratings are expressed on a four-point scale, from 0 to 3. Such ratings have been converted to binary values by considering 0-1 values as non-credible and 2-3 values as credible. Finally, we dealt with 5,509 credible and 6,736 non-credible Web pages.

### Baselines and evaluation metrics

Distinct baseline models have been taken into consideration for comparatively evaluate the effectiveness of the proposed Vec4Cred model. Traditional solutions developed for assessing the genuineness of health information, which considers both textual and metadata (handcrafted) features in association with Machine Learning, have already been tested and demonstrated their inferiority to approaches based on embedding of Web page characteristics in [58]. For this reason, in this paper we considered as baselines the original Web2Vec model (but applied to the health misinformation detection problem), the original Web2Vec model enriched with the consideration of domain-names extracted from the links present in the target Web page, and a preliminary model, of which Vec4Cred is an extension, that we proposed in [58] (such baselines are detailed below, in Section 4.3).

To assess the effectiveness of both the considered baselines and the different configurations of the proposed Vec4Cred model (also such configurations are illustrated in detail in Section 4.3), the following evaluation metrics have been taken into account: *f*1-*measure*, *accuracy*, and *Area Under the ROC Curve* (AUC). Such metrics have often been used in various literature works related to misinformation detection and information genuineness assessment [11, 34]. 5-fold stratified cross-validation has been applied in the evaluation process.

### Results

Before illustrating and discussing the results of the evaluations performed, we introduce the notations used to indicate the baselines and the configurations of Vec4Cred tested in this article, along with their details. In particular:

- Web2Vec (Baseline): it refers to the Web2Vec model applied to the health misinformation domain trained on Web page content, DOM structure and the URL of each Web page with default weight initialization;
- Web2Vec+L (Baseline): it corresponds to the previous baseline trained, in addition, by considering the word-level corpus constituted by the domain-names extracted from links present in the target Web page;
- GoodIT (Baseline): it refers to the model proposed in [58] and presented at the GoodIT 2021 Conference,^11^ trained on Web page content, DOM structure and domain-names extracted from the links present in the target Web page, with a word2vec layer trained on PubMed acting as weight initializer (i.e., the model is constituted by components (*a*) and (*c*) of the architecture illustrated in Fig. 1);
- Vec4Cred (*a*-*c*-*d*): it refers to the first configuration of the Vec4Cred model tested in this paper, which constitutes an improvement w.r.t. the GoodIT (Baseline) model, by exploiting the keywords extracted from the content of the Web pages referred from links in the target Web page. This model employs a word2vec layer trained on PubMed acting as weight initializer (i.e., the model is constituted by components (*a*), (*c*) and (*d*) of the architecture illustrated in Fig. 1);
- Vec4Cred (*a*-*c*-*e*): it refers to the second configuration of the Vec4Cred model tested in this article, which is constituted by the GoodIT (Baseline) model to which are added POS tags extracted from the target Web page content. Also this model employs a word2vec layer trained on PubMed acting as weight initializer (i.e., the model is constituted by components (*a*), (*c*) and (*e*) of the architecture illustrated in Fig. 1);
- Vec4Cred (*a*-*c*-*d*-*e*): it refers to the last configuration of the Vec4Cred model tested in this article, which combines the two above-mentioned configurations. Specifically, this model is trained on Web page content, DOM structure, the word-level corpus constituted by domain-names of the links present in the target Web page, the keywords extracted from the pages referred from such links, and the POS tags extracted from the target Web page content, with a word2vec layer trained on PubMed acting as weight initializer (i.e., the model is constituted by components (*a*), (*c*), (*d*) and (*e*) of the architecture illustrated in Fig. 1).

Concerning the three datasets considered in this article for evaluation purposes, previously illustrated in Section 4.1, they are denoted as D1 (the Microsoft Credibility Dataset), D2 (the Medical Web Reliability Corpus), and D3 (the CLEF eHealth 2020 Task-2 Dataset). In particular, only for the dataset D3, it was possible to calculate, given the higher number of labeled data, the *Binomial Proportion Confidence Intervals* with 95% confidence, as detailed in [4]. Such Binomial Intervals (BI) are reported under the label D3(BI).

Table 3 reports the results of the experimental evaluations for the considered baselines and Vec4Cred configurations, w.r.t. the three distinct datasets and the evaluation metrics taken into account. As it emerges from the table, it was not possible to evaluate the results for the D2 dataset with respect to using the first two baselines because, in the D2 dataset, the URLs of the target Web pages are not made available, as illustrated in Section 4.1.

**Table 3:** Evaluation results

		D1	D2	D3	D3(BI)
Web2Vec (Baseline)	Accuracy	80.34	–	72.31	71.32 ± 2.0
	F1	86.80	–	73.16	71.88 ± 1.7
	AUC	69.84	–	71.34	70.77 ± 1.0
Web2Vec+L (Baseline)	Accuracy	81.11	–	72.56	72.23 ± 1.0
	F1	86.78	–	73.10	71.98 ± 1.5
	AUC	78.44	–	71.11	72.00 ± 1.0
GoodIT (Baseline)	Accuracy	89.89	98.32	74.12	72.70 ± 2.0
	F1	93.78	97.01	76.61	75.00 ± 2.1
	AUC	85.69	97.71	74.56	75.40 + 1.0
Vec4Cred (*a*-*c*-*d*)	Accuracy	**90.03**	**99.05**	**80.18**	79.33 ± 2.0
	F1	**93.99**	**99.21**	**79.87**	79.01 ± 1.0
	AUC	**86.89**	**98.89**	**79.17**	78.19 ± 1.0
Vec4Cred (*a*-*c*-*e*)	Accuracy	**90.88**	**99.70**	**82.34**	81.00 ± 1.0
	F1	**94.01**	**99.41**	**82.98**	81.00 ± 1.8
	AUC	**88.27**	**99.40**	**81.01**	79.00 ± 3.4
Vec4Cred (*a*-*c*-*d*-*e*)	Accuracy	**90.47**	**99.71**	**82.56**	82.00 ± 1.3
	F1	**94.21**	**99.71**	**83.11**	82.00 ± 1.2
	AUC	**88.25**	**99.70**	**81.11**	81.00 ± 1.0

Concerning the obtained results, it is first of all possible to make an initial observation regarding the fact that all the proposed configurations of the Vec4Cred model lead to better results with respect to all three baselines considered, with respect to all three datasets and the three evaluation metrics (best results are denoted in bold). This means that the usage of an embedded representation of different Web page characteristics as illustrated in previous works, in association with a domain-specific pre-training of such embeddings on health-related data (i.e., from PubMed), and the consideration of additional genuineness-related features such as those that can be extracted from the content of related Web pages (in the form of keywords) and the POS tags in the target Web page, lead to obtain promising results w.r.t. the problem of health misinformation detection in Web pages.

If we analyze more in detail the results obtained with respect to the individual configurations tested in the Vec4Cred model, we can observe that taking into account the grammar aspects of the target Web page, through the use of POS tags in the model, i.e., Vec4Cred (*a*-*c*-*e*) makes a slightly greater contribution to the effectiveness of the model than considering the content of the linked pages within the target Web page, i.e., Vec4Cred (*a*-*c*-*d*). This emerges also by analyzing the Vec4Cred (*a*-*c*-*d*-*e*) model, whose results are not so different (even if slightly better) than those obtained for the Vec4Cred(*a*-*c*-*e*) model. This makes us think that it might be appropriate in the future, rather than simply considering the keywords extracted from the reference pages in the target Web page, to consider their grammatical structure as well, thus enriching the Vec4Cred model.

What in our opinion makes interesting these results related to the proposed model, which proves to be effective, is that it acts only by taking into account information directly extractable from the Web page, without referring to external information that could be difficult to find, such as those of the authors of the page and their role, nor additional handcrafted features as used in other approaches in the literature that have proven to be however inferior to the approach proposed already in [58]. Nevertheless, it would be interesting to be able to use some domain knowledge (where available) to test if this could further increase the effectiveness of the proposed model.

## Conclusion

In this article, we proposed Vec4Cred, a model for health misinformation detection in Web pages as an improvement of a preliminary model that we presented in [58], inspired by the Web2Vec model devoted to phishing Web page detection illustrated in [15]. The model proposed in this paper, and adapted to the considered health misinformation detection problem, is based on a multi-layer architecture, exploiting embedding representations of Web page characteristics pre-trained on health domain-specific data. With respect to [58], improvements have been proposed by considering information genuineness factors in the health domain, which can affect the identification of misinformation. Such factors, identified from the literature, are related to grammatical aspects of the target Web page and to the content of reference pages in the target Web page. In particular, embedding representations of POS tags extracted from the target Web page and keywords extracted from the pages referenced via links in the target Web page have been considered.

From the results obtained, it is possible to deduce that the various configurations of the proposed model are effective in dealing with the problem of health misinformation detection in Web pages, especially because they allow to take into account aspects related to the domain and semantics, referring only to features that can be extracted directly and automatically from the Web pages. It is possible to investigate in the future, on the basis of these results, possible improvements related to the use of contextual embedding techniques (such as BERT) and other linguistic aspects related to the pages that are referenced within the target Web page.

## Footnotes

1 In the literature, the concepts of *disinformation*, understood as false information propagated with malicious intent, *misinformation*, understood as false information generated without malicious intentions, and *malinformation*, understood as genuine information shared to cause damage, often moving information destined to remain private in the public sphere, have been introduced [60]. The definition of health misinformation that we report and employ in this article, makes no distinction between false information generated with the intent or not to cause harm.

2 The concept of “reliability” is traditionally tied to the source disseminating the information, also in the computer science scenario [23]. The concept of “credibility” takes into account distinct aspects related to the source, the message and the communication medium [35]. Although widely studied in the social sciences with subjective connotations [26, 51], it has also been applied to “computer” and Website credibility [18, 19], and recently to the automatic assessment of information genuineness circulating online [40, 59]. However, future disambiguation work remains necessary with respect to the use of these terms in the research area considered.

3 https://metacpan.org/release/WWW-Google-PageRank/

4 The HONcode certification is an ethical standard aimed at offering quality health information. https://www.hon.ch/cgi-bin/HONcode/principles.pl?English

5 JAMA is the *Journal of the American Medical Association*, a peer-reviewed medical journal published by the American Medical Association. The journal has been published since 1883, and is considered among the most authoritative in the field. https://jamanetwork.com/journals/jama.

6 DISCERN is a questionnaire designed by the UK National Health System providing users with a way of assessing the quality of information on health treatment choices. http://www.discern.org.uk/

7 https://keras.io/api/layers/core_layers/embedding/

8 PubMed is a free search engine of biomedical scientific literature from 1949 to the present. https://pubmed.ncbi.nlm.nih.gov/

9 https://www.hon.ch/en/

10 https://clefehealth.imag.fr/?page_id=610

11 http://www.grc.upv.es/goodit2021/
